# Nitramine-Group-Containing Energetic Prepolymer: Synthesis, and Its Properties as a Binder for Propellant

**DOI:** 10.3390/polym11121966

**Published:** 2019-11-29

**Authors:** Kiwon Hwang, Hyunsung Mun, Jin Young Jung, Hye Lim Cho, Sung June Kim, Byoung Sun Min, Heung Bae Jeon, Wonho Kim

**Affiliations:** 1 Department of Polymer Science & Chemical Engineering, Pusan National University, Pusan National University, Busan 609-735, Korea; kiwon8348@gmail.com (K.H.); ansehdwns10@gmail.com (H.M.); 2 Department of Chemistry, Kwangwoon University, Seoul 139-701, Korea; malinboy93@gmail.com (J.Y.J.); hachi2387@naver.com (H.L.C.); hbj@kw.ac.kr (H.B.J.); 3 Advanced Propulsion Technology Center, Agency for Defense Development, Daejeon 305-600, Korea; sungjunekim7534@add.re.kr (S.J.K.); cmskmj@hanmail.net (B.S.M.)

**Keywords:** propellant, binder, nitramine-group-containing polymer, energetic polymer, mechanical properties

## Abstract

A composite solid propellant which generates high propulsive force in a short time is typically composed of an oxidizer, a metal fuel powder and a binder. Among these, the binder is an important component. The binder maintains the mechanical properties of propellant grains and endures several thermal and mechanical stresses in the engine. Several studies have been reported for the development of energetic propellant binders for increasing the propellant′s propulsive force. While several materials have been studied for the synthesis of energetic prepolymers, a nitramine-group-containing prepolymer is a suitable candidate because these types of prepolymers are less toxic and more cost-effective when compared to the traditional glycidyl azide polymers (GAP) and triazole-based prepolymers. Considering the lack of studies for the binder using a nitramine-group-containing prepolymers, we synthesized a nitramine-group-containing monomer and polymerized a nitramine-group-containing prepolymer. The prepolymer was then used for the preparation of the binder and its thermal and mechanical properties, as well as the effect of the plasticizer, were studied. The binder that was prepared using the prepolymer containing a nitramine-group showed very high elongation, tensile strength. Nitrate-ester (NE)-type plasticizer could reduce the glassy transition temperature (*T*_g_)of the binder successfully. Also, high-energy is released due to the decomposition of the nitramine-group at around 245 °C, thus exhibiting the efficiency of the nitramine-group-containing prepolymer as an excellent energetic binder material.

## 1. Introduction

The 20th-century world wars and competitive international space exploration have contributed extensively to the development of rocket propellants, which have been continually studied for development of the aerospace industry and long-range missiles. Propellants are classified as liquid and solid propellants according to their phase. However, liquid-fuel engines, which use liquid propellant, typically have complex structures which restrict their size. In contrast, solid propellants are widely used because they allow the design of variable size fuel engines [1]. Among the various types of solid propellants available, the composite-solid propellant started with a polysulfide binder by Thiokol in the 1950s in the United States, is easy to store and can generate high propulsive force in a short time. Thus, due to its several merits, extensive research has been carried out about this composite-solid propellant [2]. 

Composite solid propellants are composed of an oxidizer, which is the solid component that supplies the oxygen needed for combustion; metal fuel-powder (energetic materials), that releases high-energy when the propellant is combusted; plasticizer, which improves processability; and a binder, that physically bonds the oxidizer and the fuel. Among them, the plasticizer is an important component of the composite-solid propellant. Plasticizer can reduce the viscosity of the mixture-paste when mixing with the binder, the oxidant and the metal fuel-powder, thereby improving processability and increasing casting time [3]. Especially, nitrate-ester (NE)-type plasticizers have been widely used for military purposes because they can improve the performance of propellants due to the NE part [3,4]. The binder can maintain the mechanical properties of propellant-grains and endures thermal and mechanical stresses present both in the inside and outside of the engine. In addition to acting as an adhesive which helps in bonding the propellant to the motor case, the binder also acts as combustible material which is required for generating propulsive force during combustion [5]. 

A rubber-like viscoelastic material obtained by the reaction of a low-molecular-weight polymer (prepolymer) with a curing agent is typically used as the binder material. Generally, the prepolymer used in binders for the preparation of composite solid propellants is a low-molecular-weight polymer which is amorphous and exhibits flowability and forms a polymer network structure through crosslinking. Among various types of crosslinking binders, such as polyethylene glycol (PEG) in the form of polyether or polyester, hydroxyl-terminated polybutadiene (HTPB) and polycaprolactone (PCL) are widely used in the preparation of propellants [6,7,8,9]. 

In recent years, the development of high-energy propellant binders has been actively carried out for increasing the propulsive force of the propellant. Usually, glycidyl azide polymers (GAPs), triazole-crosslinked polymers and nitramine-group-containing polymers have been studied as energetic prepolymer candidates. GAP is an energetic polymer which releases a large amount of gas and thermal energy during combustion and is widely used in propellants [10,11]. Min et al. [12] had studied the physical properties of a polyurethane binder prepared with GAP and further studies [13] were conducted for the evaluation of the physical properties of the binder which was prepared with a polyurethane and triazole-dual-crosslinked system. However, GAP is expensive and its other disadvantages include the necessity for the use of toxic and explosive monomers for its synthesis and its tendency to releases gases during processing or storage [14].

A triazole-crosslinked polymer, which has been systematically studied by Huisgen [15], is a polymer containing a triazole ring formed by a “click reaction.” Due to their moisture insensitivity and the lack of by-product formation, triazole crosslinked polymers are attracting attention as eco-friendly energetic materials [16,17,18,19]. Lee et al. [20] studied the properties of binders with a triazole curing system to assess their impact on the properties of the binders. Furthermore, the effects and behavior of plasticizers in the triazole-crosslinked polymer were also studied [21]. While triazole-crosslinked-polymers exhibited excellent performance characteristics, their poor cost-competitiveness by the relatively expensive raw materials required for the synthesis are the major disadvantages for their use.

To solve these kinds of problems, a relatively cheap and less toxic nitramine group (–N–NO_2_) was introduced into the polymer backbone and the synthesis of polyether and polyester-type polymer containing the nitramine group has been reported [22,23]. A polymer containing a nitramine-group typically shows proper viscosity and glass transition temperature and is resistant to hydrolysis. However, studies on the preparation of a binder introducing this group and a study of its physical properties have not been carried out yet [24]. The effect of the nitramine-group on the binder was usually confirmed by studies which added a cyclic nitramine, such as hexahydro-1,3,5-trinitro-1,3,5-s-triazine (RDX) or 1,3,5,7-tetranitro-1,3,5,7-tetraazacyclooctane (HMX), to the binder [25,26,27], or studies which used a polymer containing a nitro group that is similar to the nitramine group [28,29]. Thus, studies exploring the properties of binders which use a nitramine-group-containing polymer have been lacking.

Thus, in this study, a nitramine group-containing prepolymer was synthesized by using a monomer containing the nitramine group in their molecular structure to investigate the effect of the nitramine group present in the polymer backbone of the binder on the propellant. The binder was then prepared by using the synthesized low-molecular-weight energetic prepolymer and its thermal and mechanical properties were studied. Additionally, the effect of plasticizer on binder was studied by using binder which was applied NE-type plasticizer.

## 2. Experimental Section

### 2.1. Materials for Monomer Synthesis and Prepolymer Polymerization

For monomer synthesis, ethylenediamine (≥99%) was purchased from Merck, Kenilworth, New Jersey, USA. Acrylonitrile (>99.0%) was purchased from Tokyo Chemical Industry (TCI), Nihonbashi-honcho, Japan, and hydrochloric acid (35.0%) was purchased from DAEJUNG Chemicals, Korea. Acetic anhydride (>93%) and methyl alcohol (>99.8%) were purchased from DUKSAN Chemicals, Daejeon, Korea, and nitric acid (95%) was purchased from SAMCHUN Chemicals, Seoul, Korea. For the polymerization of the prepolymer, diethylene glycol (DEG) (>99.5%) was purchased from Tokyo Chemical Industry (TCI), Nihonbashi-honcho, Japan.

### 2.2. Materials for Binder Preparation and Plasticizer

Trifunctional group curative (Desmodur N-3200) and the TPB (triphenyl bismuth) catalyst which were required for the preparation of the binder, were purchased from Covestro and DONGIN Chemicals, respectively. The poly (diethylene glycol adipate), a polyester-type prepolymer synthesized with adipic acid and diethylene glycol, which was used for comparison with the energetic binder, was purchased from SONGWON Industrial Co., Korea. And for controlling *T*_g_ of binder, the nitrate-ester (NE)-type plasticizer, butanetriol trinitrate (BTTN) and trimethy-lolethane trinitrate (TMETN), were supplied by Agency for Defense Development, Korea.

### 2.3. Synthesis of Energetic Monomer

Acetic anhydride (94 mL) was placed in a 2-neck flask and was cooled to 0 °C under an argon atmosphere. Nitric acid (95%, 2.7 g, 0.04 mol) and hydrochloric acid (35%, 1.32 g, 0.04 mol) were added to the cooled flask. The resulting mixture (3,3′-(ethane-1,2-diylbis(azanediyl))dipropanenitrile nitric acid salt (19.90 g, 0.068 mol)) was added by portionwise at 35 °C for 2 h and was then stirred for 4 h at 35 °C. A solution of 35% hydrochloric acid (3.00 g, 0.08 mol) was added to the reaction mixture until the reactant turned dark yellow. The reaction mixture was stirred at 35 °C for 30 min, the temperature was increased to 55 °C and the stirring was continued for another 30 min. The temperature was then cooled to 10 °C and the reaction mixture was crystallized by addition of cold distilled water (120 mL). The crystallized solid was filtered off under reduced pressure to obtain *N*,*N*′- (ethane-1,2-diyl) bis (*N*-(2-cyanoethyl) nitramide) (8.80 g, 50%) as a white solid.

**Figure 1:**
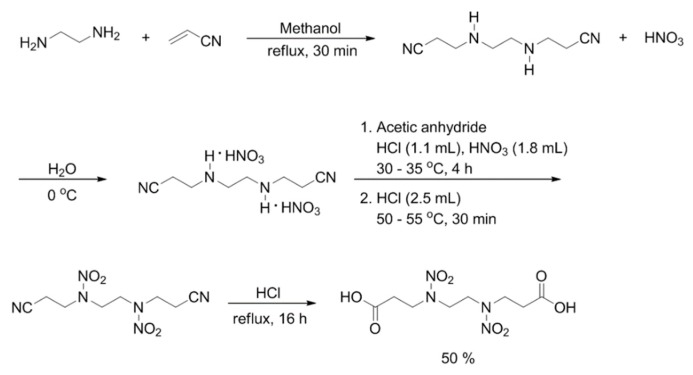
Synthesis of 4,7-dinitrazadecanoic-1,10-diacid.

Hydrochloric acid (35%, 160 mL, 5.26 mol) was added to the *N*,*N*′- (ethane-1,2-diyl) bis (*N*-(2-cyanoethyl) nitramide and the mixture was refluxed for 16 h. After removal of water by vacuum distillation, the product was cooled and recrystallized at 5 °C and was filtered off under reduced pressure. The product was dried to obtain 4,7-dinitrazadecanoic-1,10-diacid (DNDA) (10 g, 99%) as a white solid and the DNDA monomer melting point is 100 °C. A brief synthesis of the monomers is shown in Figure 1.

### 2.4. Polymerization of Energetic Prepolymer

**Figure 2:**
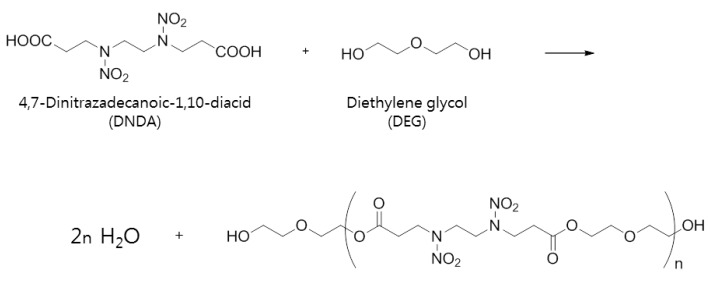
Polymerization of energetic prepolymer.

Diethylene glycol was reacted with the carboxyl groups at both ends of the synthesized energetic monomer 4,7-dinitrazadecanoic-1,10-diacid for polymerization. Figure 2 shows the condensation of the carboxyl group at both ends of the monomer with the alcohol, which results in the formation of ester linkages. As both reactants are highly reactive, the ester-type polymer was easily obtained by mixing and heating the 4,7-dinitrazadecanoic-1,10-diacid and diethylene glycol. The 4,7-dinitrazadecanoic-1,10-diacid (1.27 g, 0.0043 mol) was reacted with diethylene glycol (0.73 g, 0.0069 mol) in the presence of the p-toluenesulfonic acid (0.013 g, 0.00008 mol) catalyst. During the reaction, nitrogen gas was continuously purged through the flask and the reaction mixture was stirred at 120 °C for 8 h while removing water. The product was dissolved in CH_2_Cl_2_ and was precipitated in MeOH. After drying at 60 °C for 12 h in a vacuum oven, the prepolymer (DNDA_DEG, 1.8 g, 90%) containing the –OH group at the terminal was synthesized.

### 2.5. Characterization of Energetic Monomer and Prepolymer

^1^H- and ^13^C-NMR spectra were acquired at 400 and 100 MHz using a JNM-AL400 spectrometer (JEOL, Japan), respectively. The number-average molecular weight (*M*_n_) and molecular weight distribution (MWD) were determined using GPC (gel permeation chromatography) calibrated with poly ethylene glycol (PEG) standard. The GPC consisted of Agilent 1100 pump, Refractive Index Detector and PSS GRAM (5 μm, 102 Å 8.0 × 300.0 mm) columns.

Fourier-transform infrared spectroscopic (FT-IR) analysis was carried out using a Nicolet 6700 spectrometer (Thermo Scientific) at a resolution of 8 cm^−1^ in the 650–4000 cm^−1^ spectral range using an attenuated total reflection (ATR) method.

The OH value of the prepolymer was measured using an auto titrator (Metrohm 888 Titrando Model) according to ASTM E 1899-08 after titration with tetrabutylammonium hydroxide (TBAOH).

### 2.6. Formation of Urethane Crosslinked Binder Network

A prepolymer containing an –OH group at the terminal position, plasticizer, and Desmodur N-3200, a trifunctional group curative containing the NCO group, were mixed at the functional ratio of 1:1.1 (–OH/–NCO). DNDA_DEG prepolymer is high viscosity liquid phase. It is possible to manufacture the binder at room temperature, but this mixing process was carried out at 60 °C to improve processability. A solution of the TPB in acetone was added to this mixture as a catalyst and the mixture was stirred for 15 min. The acetone was removed by an evaporator with stirring at 60 °C for 30 min. The resulting compound was poured into a mold and curing was performed at 60 °C for one week.

### 2.7. Thermogram Analysis

Thermal properties of the polymer were measured with a differential scanning calorimeter (Instrument DSC Q20 V24.11 Build 124) at a −10 °C/min rate under a nitrogen atmosphere at −80 to 100 °C. The mass loss was measured in the 10 to 510 °C range under a nitrogen atmosphere at a rate of 10 °C/min by the thermogravimetric analysis (TGA 550, TA Instruments).

### 2.8. Mechanical Properties

The mechanical properties were measured using a universal testing machine (UTM; KSU-05M-C, KSU Co., Ansan, Korea) with a sample of dimensions 35 mm (length) × 6 mm (width) × 1 mm according to ISO-37 type 4. The elongation and tensile strength were measured at a rate of 50 mm/min at room temperature.

## 3. Results and Discussion

### 3.1. Characterization of Energetic Monomer and Prepolymer

#### 3.1.1. H-NMR and ^13^C-NMR Spectra of Energetic Monomer

**Figure 3:**
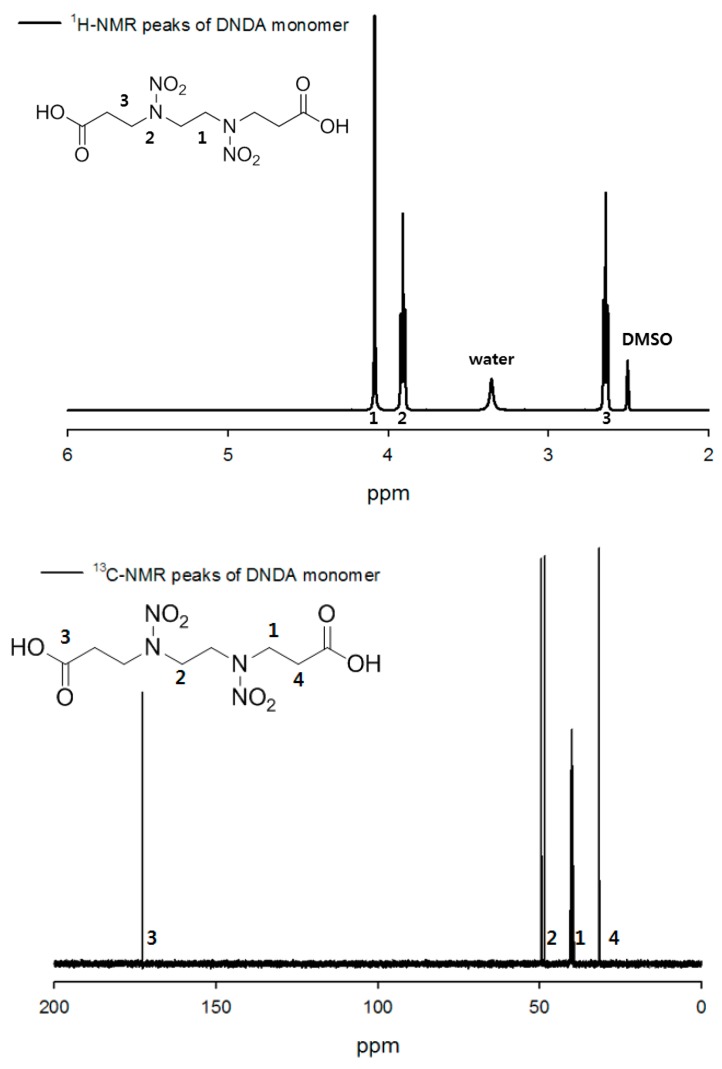
^1^H-NMR and ^13^C-NMR spectra of 4,7-Dinitrazadecanoic-1,10-diacid (DNDA) monomer.

The ^1^H- and ^13^C-NMR spectra of the prepared 4,7-dinitrazadecanoic-1,10-diacid were acquired for confirming the chemical structure (Figure 3). 4,7-Dinitrazadecanoic-1,10-diacid: white powder; ^1^H NMR (400 MHz, DMSO-d6) δ 4.08 (s, 4 H), 3.91 (t, *J* = 8.0 Hz, 4 H), 2.63 (t, *J* = 4.0 Hz, 4 H). ^13^C-NMR (100 MHz, DMSO-d6) δ 172.2, 48.9, 47.8, 31.0.

#### 3.1.2. H-NMR Spectrum of Energetic Prepolymer

**Figure 4:**
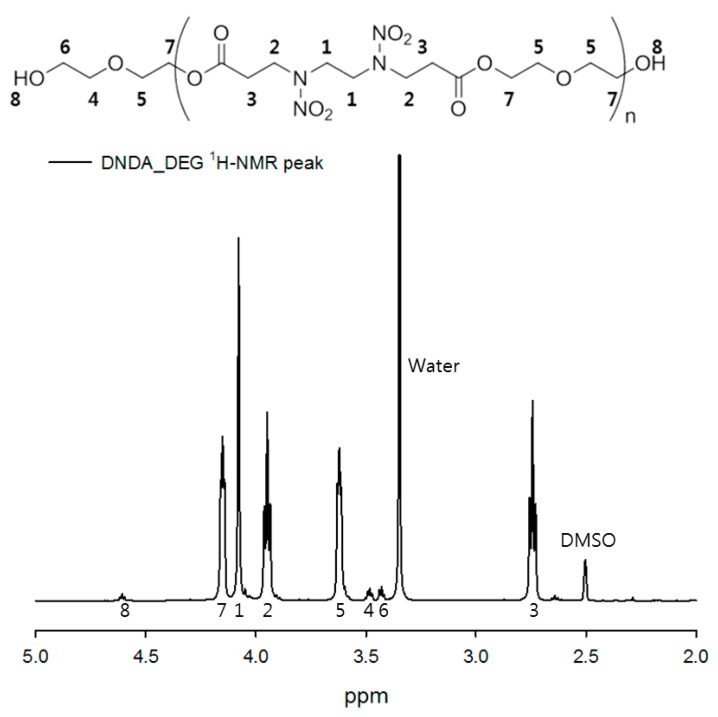
^1^H-NMR spectrum of DNDA_DEG prepolymer.

A GPC measurement of the polymer resulting from the reaction of DNDA, the monomer-containing nitramine group, with diethylene glycol, showed that the prepolymer had a number-average molecular weight (*M*_n_) of 2300 g/mol and a molecular weight distribution (MWD) of 1.5. ^1^H-NMR data showed the microstructure of the synthesized prepolymer. The –OH group at the end of the prepolymer showed a peak at 4.62 ppm and the presence of the nitramine-group (–N–NO_2_) was confirmed by the peaks at 3.95 and 4.08 ppm. The peak at 2.75 ppm confirmed the formation of the ester group by condensation polymerization and the peaks at 4.15 and 3.62 ppm indicated the presence of the glycol-polymerized polymer backbone. The calculation of the area-ratio confirmed that a low-molecular-weight prepolymer having five to six repeating units was synthesized. Detailed results are shown in Figure 4.

#### 3.1.3. FT-IR Spectrum of Energetic Prepolymer

**Figure 5:**
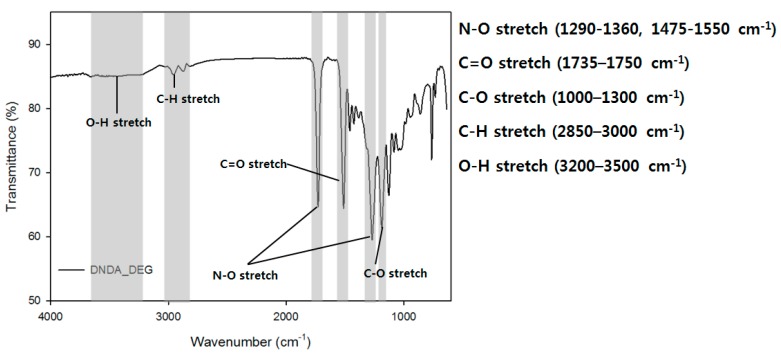
DNDA_DEG prepolymer FT-IR spectra.

FT-IR measurement results of the prepared prepolymer are shown in Figure 5. The broad peak at 3200–3500 cm^−1^ indicated the presence of the –OH group at the terminal position of the prepolymer. The peaks at 1735–1750 cm^−1^, indicated the presence of the carbonyl group of the ester and the peak at around 1290–1360 cm^−1^ indicated the presence of the –NO_2_ group. Thus, the IR analysis confirmed the synthesis of a prepolymer having a nitramine group and a terminal –OH in the polymer chain.

#### 3.1.4. Thermogravimetric Analysis of Monomer and Prepolymer

The thermal properties of the monomer were measured and show the melting point at 113.4 °C. The thermal properties of the commercial SS-207 prepolymer and that of the synthesized DNDA_DEG prepolymer were compared. The SS-207 prepolymer, used as a reference, is a polyester-type prepolymer synthesized with adipic acid and diethylene glycol and has a similar molecular structure to that of the DNDA_DEG prepolymer.

**Figure 6:**
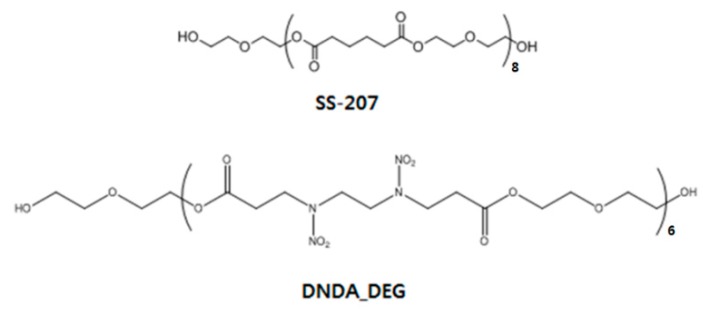
Structure of prepolymers used for preparing binder.

**Figure 7:**
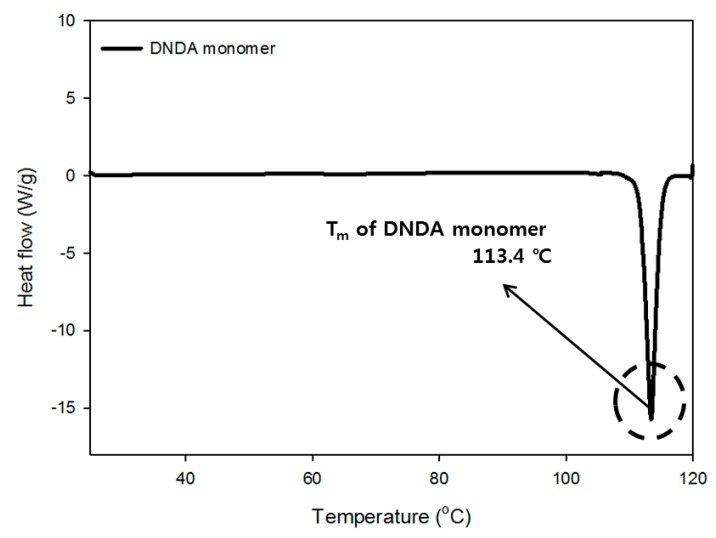
DSC thermogram of DNDA monomer.

**Figure 8:**
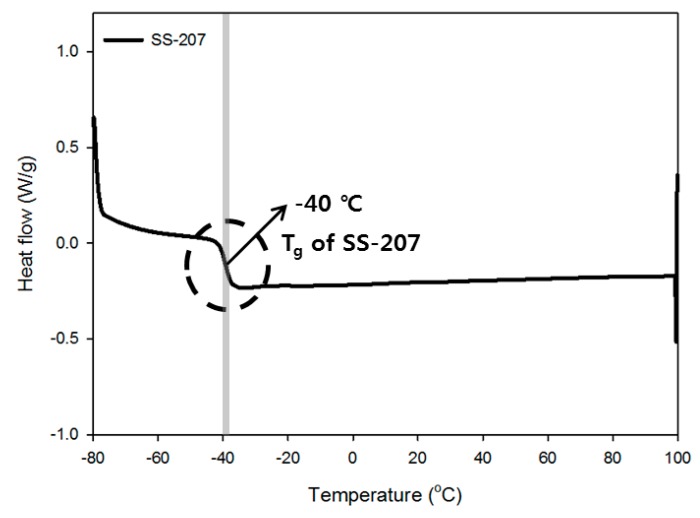
DSC thermogram of SS-207 prepolymer.

**Figure 9:**
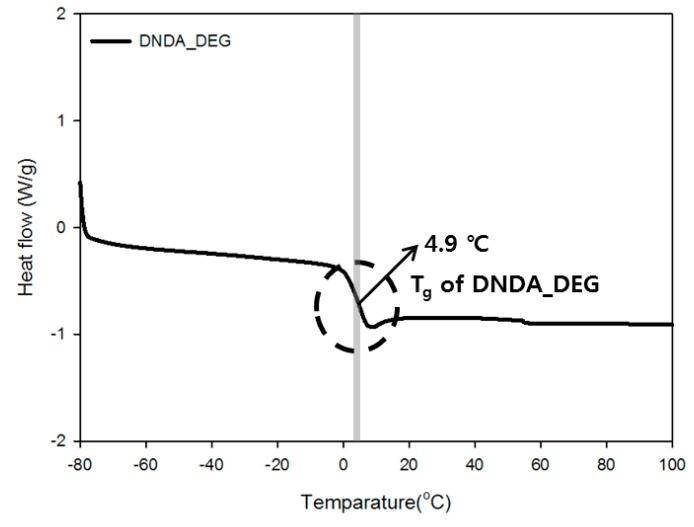
DSC thermogram of DNDA_DEG prepolymer.

The cohesive energy density, which indicates the agglomeration characteristics of the polymer chain, of the synthesized DNDA_DEG prepolymer, was high due to the presence of the energetic nitramine group and which was also reflected by its higher *T*_g_ than that of the SS-207 [30,31,32]. Furthermore, the formation of an amorphous polymer was confirmed by the lack of the change in the direction of the heat flow up to 100 °C. Prepolymer chemical structures that we use are shown in Figure 6 and detailed DSC thermograms are shown in Figure 7, Figure 8 and Figure 9.

### 3.2. Formation of Urethane Crosslinked Binder Network

An energetic binder was prepared using the DNDA_DEG prepolymer containing the nitramine group and a comparative evaluation was conducted with a binder which did not contain a nitramine group. A DNDA_DEG prepolymer which had a molecular weight similar to that of SS-207 was synthesized and the –OH value was measured before the preparation of the binder. The binder was prepared using DNDA_DEG prepolymer and Desmodur N-3200, a trifunctional curing agent, based on the measured –OH value. Additionally, binders are prepared using NE-type plasticizer to solve the high *T*_g_ problem of DNDA_DEG prepolymer. The detailed binder formulations are shown in Table 1 and confirm that a clear transparent elastomer was formed by the crosslinking of the binder.

### 3.3. Thermogravimetric Analysis of Binders

**Figure 10:**
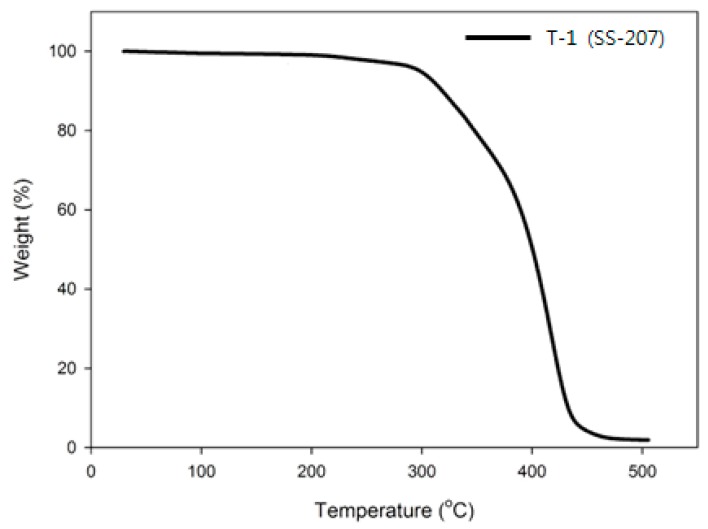
TGA thermogram of SS-207 binder.

**Figure 11:**
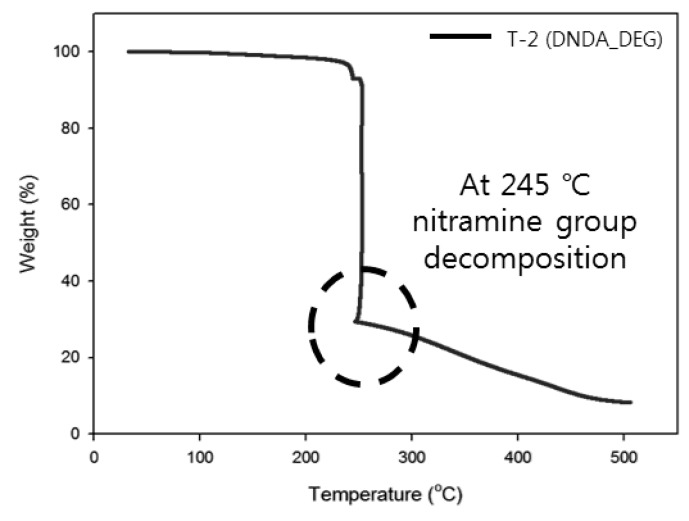
TGA thermogram of DNDA_DEG binder.

The thermal properties of the prepared binder were measured using DSC and TGA and the detailed DSC results are shown in Table 2, also TGA results are shown in Figure 10 and Figure 11. In the case of the T-2 compound, which was prepared by using a prepolymer containing the nitramine group, shows higher *T*_g_ than T-1 compound due to *T*_g_ of DNDA_DEG prepolymer. So, NE-type plasticizer was applied to reduce the *T*_g_ of the binder prepared by using DNDA_DEG prepolymer, and effectively lowered the *T*_g_ of the binder and shows lower *T*_g_ than T-1 compound when more than 200 wt % of plasticizer, compared to prepolymer, was applied. 

In the case of the T-1 compound, which was prepared by using a commercial prepolymer, the backbone of the polymer began to decompose at around 300 °C according to the TGA results and the continuous mass reduction was observed up to 450 °C, which is similar to the thermal degradation behavior of the general polymer. However, unlike the T-1 compound, the T-2 compound prepared by using the prepolymer containing the nitramine group showed a rapid decrease of mass due to the decomposition of the nitramine group at 245 °C. Therefore, in the case of the T-2 compound, it can be confirmed that the nitramine group exists in the urethane network after the preparation of binder and a larger propulsive force can be obtained at a lower temperature using this material than with the use of the binder prepared using the commercial prepolymer.

### 3.4. Mechanical Properties of Binders

Since the DSC measurement showed the amorphousness of the prepared binder, mechanical properties were measured at room temperature. The T-2 compound prepared from prepolymer containing a nitramine-group showed four-times higher tensile strength and five-times higher elongation than the T-1 compound prepared with the commercial prepolymer. Generally, the presence of an energetic functional group such as an azido group in the polymer backbone affects the mechanical strength and elongation of the binder [5,33]. Even though the T-2 compound forms a urethane network similar to that of the T-1 compound, the internal energy of the T-2 compound is improved due to the presence of the nitramine group in prepolymer backbone. 

In the case of the compound that the plasticizer is applied, the tensile strength and the elongation decreases as the content of the plasticizer increases. Generally, studies have reported that the crosslinking density is significantly affected by the plasticizer. As a result of the addition of plasticizer, tensile strength and elongation showed decreases. [34,35] Especially in the case of compound with 300 wt % of plasticizer compared to prepolymer, the amount of plasticizer is high, so it is difficult to form polymer network, so showed worse mechanical properties.

**Figure 12:**
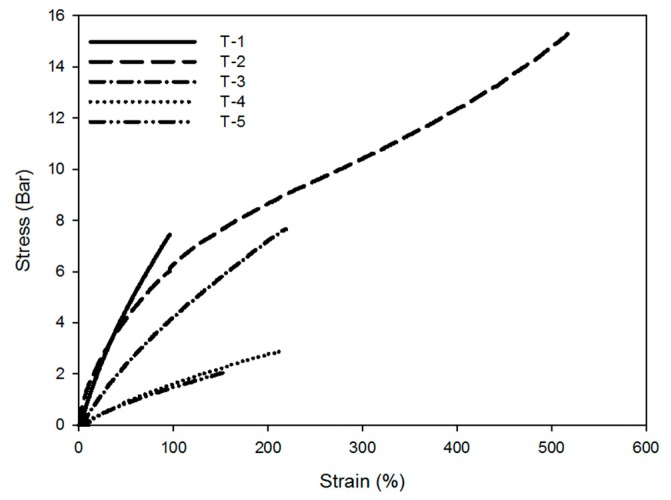
Mechanical properties of binder.

However, T-3 and T-4 compounds were found to have low *T*_g_ while showing excellent mechanical properties as a good solid propellant binder. (tensile strength >2 bar, elongation at break >200%, and *T*_g_ < −20 °C) Accordingly, high-energy binder with excellent mechanical properties can be prepared by using prepolymer containing the nitramine group synthesized in this laboratory and urethane network binder having a low *T*_g_ can be prepared by applying plasticizer of NE type. The detailed results are shown in Figure 12 and Table 3.

## 4. Conclusions

The purpose of this study was to investigate the effect of a nitramine group present in the polymer backbone on the properties of the binder for the propellant. We synthesized a low-molecular-weight high-energetic prepolymer with a monomer containing the nitramine group in the molecule and, using this prepolymer, we prepared a binder and studied its thermal and mechanical properties. The experimental conditions for the synthesis of the energetic monomer containing the nitramine group were established and the monomer was used for the polymerization of a polyester-type nitramine-containing prepolymer with a terminal –OH group. The structures of the synthesized energetic monomer and the energetic prepolymer were confirmed by ^1^H- and ^13^C-NMR and the presence of the nitramine group was confirmed through FT-IR analysis. Importantly, the synthesized prepolymer is a stable amorphous polymer at 60 °C, which is the condition used for preparing the binder.

Notably, the thermal characterization of energetic binder showed a rapid weight loss due to the decomposition of the nitramine group at 245 °C. Furthermore, through this study, the applicability of the nitramine-group-containing prepolymer as a propellant binder was confirmed and the effect of the nitramine group on the polymer backbone for improving the mechanical properties of the polyurethane binder was established. The polyurethane binder prepared by using the NE-type plasticizer, polyester-type prepolymer containing the nitramine group showed excellent mechanical properties (tensile strength >2 bar, elongation at break >200%, and *T*_g_ < −20 °C) as a composite-solid propellant binder.

Since the evaluation of binders using nitramine-group-containing prepolymer in various curing systems such as polysulfides and triazoles has not been studied yet, this study will facilitate the development of such applications and will allow the synthesis of efficient energetic binders for propellants.

## Figures and Tables

**Table 1:** Formulation of urethane-crosslinked binder network.

Step	Unit	T-1	T-2	T-3	T-4	T-5
Prepolymer –OH value	-	SS-207	DNDA_DEG			
	mgKOH/g	56	56	56	56	56
	mole/kg	1	1	1	1	1
Curatives –NCO value	mole/kg	5.45	5.45	5.45	5.45	5.45
–NCO: –OH	-	1.1	1.1	1.1	1.1	1.1
Prepolymer	g	2.909	2.909	1.588	1.092	0.832
Curatives	g	0.587	0.587	0.321	0.220	0.168
Catalyst	g	0.004	0.004	0.004	0.004	0.004
Plasticizer	g	-	-	1.588	2.184	2.496

**Table 2:** DSC data of urethane crosslinked binder network.

Title	T-1	T-2	T-3	T-4	T-5
*T*_g_ (°C)	−40	5.5	−26	−44	−49

**Table 3:** Mechanical properties of the binder.

Title	Unit	T-1	T-2	T-3	T-4	T-5
Elongation at break	%	96	517	219	211	157
Tensile strength	bar	7.4	15.3	7.6	2.9	2.1
